# Effects of Acute MDMA Intoxication on Mood and Impulsivity: Role of the 5-HT_2_ and 5-HT_1_ Receptors

**DOI:** 10.1371/journal.pone.0040187

**Published:** 2012-07-10

**Authors:** Janelle H. P. van Wel, Kim P. C. Kuypers, Eef L. Theunissen, Wendy M. Bosker, Katja Bakker, Johannes G. Ramaekers

**Affiliations:** Department of Neuropsychology and Psychopharmacology, Faculty of Psychology and Neuroscience, Maastricht University, Maastricht, the Netherlands; California Pacific Medicial Center Research Institute, United States of America

## Abstract

MDMA induces positive mood and increases impulse control during intoxication, but only a few studies on the neuropharmacological mechanisms underlying these processes have been conducted. It was hypothesized that pretreatment with 5-HT_1_ and 5-HT_2_ receptor blockers would prevent MDMA effects on mood and impulsivity. Subjects (N = 17) participated in a double-blind, placebo controlled, within-subject design involving 6 experimental conditions consisting of pretreatment (T1) and treatment (T2). T1 preceded T2 by 30 minutes. T1–T2 combinations were: placebo-placebo, 20 mg pindolol-placebo, 50 mg ketanserin-placebo, placebo-75 mg MDMA, 20 mg pindolol-75 mg MDMA and 50 mg ketanserin-75 g MDMA. Subjects completed a Profile of Mood States (POMS) questionnaire and several impulsivity tasks (Stop signal task, Matching familiar figures task, Cue dependent reversal learning task) at 1.5 hrs post-treatment. MDMA alone increased both positive (vigor, arousal, friendliness, elation, positive mood) and negative affect (anxiety, confusion) as assessed by the POMS questionnaire. MDMA also increased stop reaction time in the Stop signal task and reaction time in the Matching familiar figures task. Pretreatment with ketanserin blocked MDMA effects on positive affect, but not negative affect. Ketanserin did not influence the effects of MDMA on impulsivity. Pindolol did not interact with MDMA on any of the measures. In conclusion, 5-HT_2_ receptors mediate positive moods induced by MDMA but not negative moods or impulsivity. 5-HT_1_ receptors do not appear to be involved in MDMA effects on mood and impulse control.

**Trial Registration:**

Nederlands Trial Register NTR2352

## Introduction

3,4-Methylenedioxymethamphetamine (MDMA) is a serotonin (5-HT) agonist and a reuptake inhibitor of serotonin and dopamine (DA) that has been shown to affect mood [1] and impulsivity during intoxication [2], [3], [4] and abstinence [5], [6]. Mood has been shown to be affected by fluctuations in 5-HT levels. High levels of 5-HT have been associated with mood elevations, while decrements in 5-HT are associated with depressed mood [7], [8], [9]. MDMA has been shown to increase 5-HT levels during intoxication but to deplete 5-HT after intake [8], [9], [10], [11]. Vollenweider and colleagues [4] discuss a series of studies in which a single dose of MDMA was shown to directly increase subjective feelings of openness, enhance mood and well-being and heighten feelings of happiness. Other researchers also reported that MDMA increased subjective ratings of feeling ‘loving’ and ‘friendly’, as indicated by the Profile of Mood States (POMS) questionnaire [12]. However, depressed mood has been reported in MDMA-users following acute administration of the drug, probably due to a depletion of 5-HT stores [13], [14], [15].

A similar relation may hold true for MDMA effects on impulsive behaviors. A reduction of 5-HT has been linked to impulsive, suicidal and aggressive behaviour [16] and substance abuse [17]. In contrast, high levels of 5-HT have been shown to decrease impulsive behaviour [16], [18]. Likewise, acute MDMA administration has been shown to increase impulse control, when 5-HT levels are high [1], [2], [3], [19], whereas impulse control decreased in the period following use, when 5-HT levels are low [6], [20], [21], [22], [23], [24], [25].

Besides general 5-HT levels, there is also evidence that 5-HT_1_ and 5-HT_2_ receptors contribute to the influence of MDMA on mood and impulsivity [26]. Liechti and Vollenweider [27] have shown that a 5-HT_2_ receptor antagonist (ketanserin) decreased the effects of MDMA on perception and emotional excitation. These investigators also demonstrated that a 5-HT_1_ blocker (pindolol) ameliorated some, but not all, of the MDMA-induced subjective effects [28]. The role of 5-HT_1_ and 5-HT_2_ antagonists on MDMA effects in impulsivity has not been studied before. However it has been shown that these receptors may play a role in impulse control. For example, 5-HT_2_ antagonists have been shown to decrease impulsivity [29] whereas 5-HT_1_ agonists have been associated with reductions in anxiety and impulsivity [30].

The present study was designed to assess the role of 5-HT_1_ and 5-HT_2_ receptors on the effects of MDMA on mood and impulse control. It was hypothesized that (1) an acute dose of MDMA would alter mood and increase impulse control; and that (2) the effects of MDMA on mood and impulsivity would be absent when pre-treated with 5-HT_1_ and 5-HT_2_ receptor blockers.

## Materials and Methods

### Subjects

Seventeen healthy MDMA-users (9 male, 8 female), aged between 19 and 27 (mean (SD) 22.76 (2.75)) years participated in the study. They were mild to moderate users of MDMA who reported to have taken the drug on 1 to 65 separate occasions (mean 10.94) in the previous year. Overall, subjects reported to have taken MDMA 3 to 780 occasions in their lifetime (mean 72.4 times). Subjects reported mean lifetime use of alcohol on 1168.5 occasions; cannabis on 554.4 occasions; amphetamines on 4.1 occasions; cocaine on 4.7 occasions; LSD on 4.4 occasions; mushrooms on 0.2 occasions; and other drugs, including but not limited to, khat, ketamine or salvia divinorum, 0.5 occasions.

Subjects were recruited through advertisements in local newspapers and by word of mouth. Before inclusion, subjects were examined by a medical supervisor, who checked vital signs and took blood samples for standard blood chemistry and haematology. Inclusion criteria were: written informed consent; age 18–35 yr; history of MDMA use; free from psychotropic medication; good physical health as assessed by a medical doctor; normal weight as determined by BMI 18–28. Exclusion criteria were: addiction according to DSM-IV criteria as assessed by a questionnaire; presence or history of psychiatric or neurological disorder as assessed during a clinical interview; pregnancy or lactating; cardiovascular abnormalities; excessive drinking or heavy smoking, i.e. defined as more than 20 standard units of alcohol per week and more than 10 cigarettes per day; hypertension. Subjects were given an information leaflet before the study explaining the entire study procedure. Subjects were fully aware of everything that would happen during the study, except the order of treatment.

This study was conducted according to the code of ethics on human experimentation established by the declaration of Helsinki (1964) and amended in Seoul (2008) and was approved by the Medical Ethics Committee of the Academic Hospital of Maastricht and Maastricht University. A permit for obtaining, storing and administering MDMA was obtained from the Dutch drug enforcement administration. Subjects were paid for their participation in the study.

### Design, Doses and Administration

Subjects participated in a double-blind, placebo controlled, within-subject design involving 6 experimental conditions consisting of pretreatment (T1) and treatment (T2). T1 preceded T2 by 30 minutes. T1–T2 combinations were: placebo-placebo, 20 mg pindolol-placebo, 50 mg ketanserin-placebo, placebo-75 mg MDMA, pindolol 20 mg-MDMA 75 mg and 50 mg ketanserin-75 mg MDMA. Conditions were separated by minimum wash-out period of 7 days to avoid cross-condition contamination. Order of conditions was divided in 3 blocks of 6 conditions. The 75 mg dose of (racemic) MDMA was selected because it falls within the normal range of recreational use [24] and has been consistently shown to impair performance and produce robust subjective mood changes in a number of previous studies from our group [31], [32], [33]. Doses of pindolol 20 mg and ketanserin 50 mg represent regular therapeutic doses that block approximately 40% of 5-HT_1A_ receptors and 91% of 5-HT_2_ receptors respectively [34], [35], [36]. MDMA, pindolol and ketanserin were acquired through the local hospital pharmacy, which also performed randomization, capsulation and distribution of study drugs.

### Procedures

All subjects received a training session before onset of the experimental sessions in order to familiarize them with the tests and procedures. Subjects were asked to refrain from drugs at least a week before the start of the experiment and during the study. Subjects were not allowed to use alcohol on the day prior to an experimental session and were requested to arrive at experimental sessions well rested. Drug and alcohol screens were performed upon arrival of the subject. Drug screens assessed the presence of benzodiazepines, opiates, cocaine, marijuana, MDMA and (meth)amphetamine. A pregnancy test was performed for the female subjects. Study treatments were only administered if subjects tested negative for drugs, alcohol and pregnancy.

**Figure 1:**
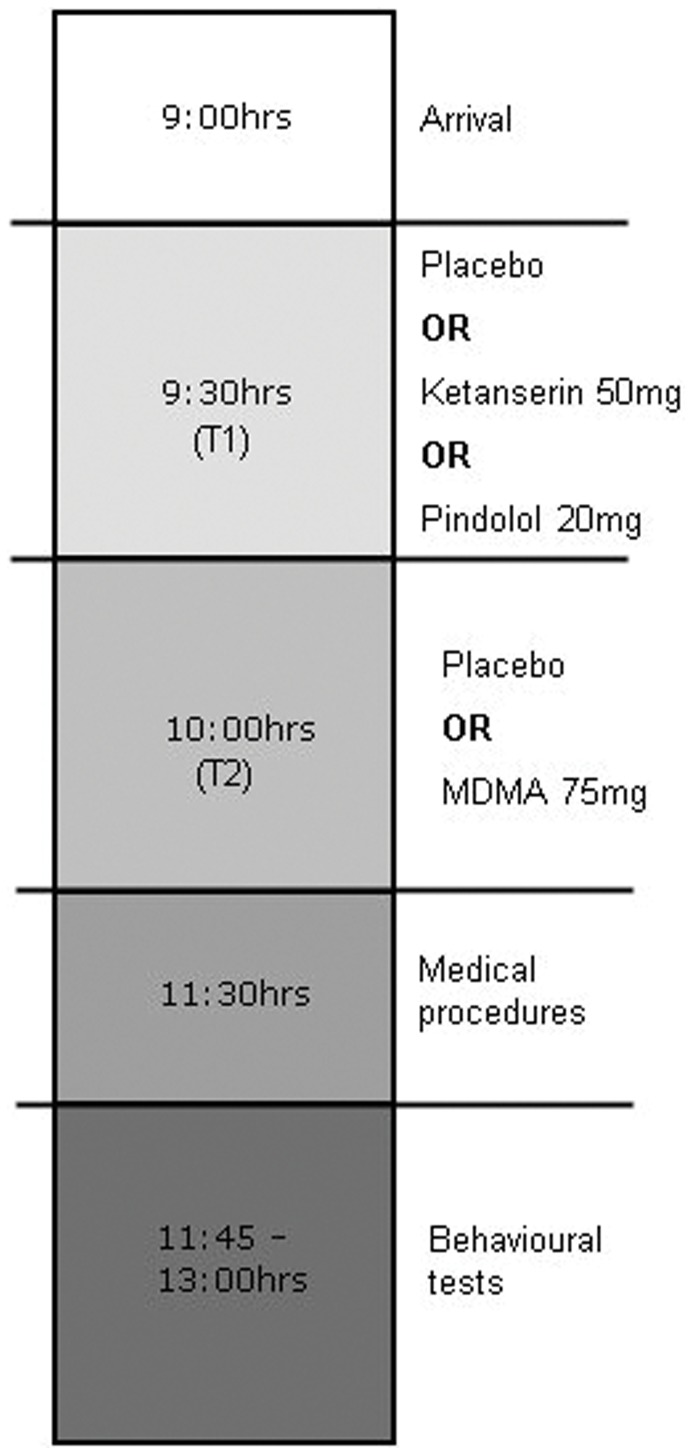
Schematic representation of a testing day.

Treatments at T1 and T2 were administered using a double dummy technique to synchronize time of maximal drug concentrations (T_max_) and were administered as identical encapsulated tablets to ensure blinding. Mood and impulsivity were assessed by means of a number of tasks between 1.5–2 hrs after T2 (at T_max_). In between, subjects were allowed to read a book or watch television. In addition, blood pressure and body temperature were assessed as safety measures (more details about physiological measures are described in a previous paper by van Wel et al. [37]). A schematic representation of a testing day is shown in figure 1.

### Subjective Measures

#### Profile of Mood States

The Profile of Mood States (POMS) is a self-assessment mood questionnaire with 72 items, rated on a 5-point Likert scale, with 0 being ‘not at all’ to 4 ‘extremely’. Subjects had to indicate to what extent these items were representative of their mood at that moment in time. Eight mood states are classified and quantified by calculating the sum score of associated items for each mood state, i.e., anxiety (9 items), depression (15 items), anger (12 items), vigor (8 items), fatigue (7 items), confusion (7 items), friendliness (8 items) and elation (6 items). Two composite scales were derived; arousal ((anxiety+vigor)−(fatigue+confusion)) and positive mood (elation−depression) [38].

### Impulsivity Tests

#### Matching Familiar Figures Task

The computerized version of the Matching Familiar Figures Test (MFF20) was derived from the original Matching Familiar Figures Test [39] by Cairns and Cammock [40] and serves as a measure of reflection impulsivity. This is the tendency to reflect on the validity of a problem to be solved under the specific condition that there are more alternatives available while there is some uncertainty over which is the right choice. Thus, subjects who tend to respond more impulsively are faster, but make more errors, while those who pause and think about the alternatives are slower and more accurate. This task was developed to assess the processes involved in the gathering and evaluation of perceptual information required to make a response. The MFF20 seems to share some variability with other impulsivity tasks, such as the stop-signal task, but not with tasks involving reward [41].

The test format of the computerized MFF20 involves simultaneous presentation of a target figure positioned on the left of the screen and an array of six alternatives on the right half of the screen, all except one differing in one or more details from the target figure. The subjects are asked to select from the alternatives the figure that exactly matches the target as quickly as possible. This is achieved by pressing the number corresponding to the figure on a computer keyboard. If the initial selection is incorrect, this is signaled with a beep and subjects are required to give another answer. Each subject is given 2 practice trials followed by 20 test trials.

Two dependent measures, mean latency to first response and total number of errors, are automatically recorded. Two additional dependent variables can be calculated: an Impulsivity score (I-score) and an Efficiency score (E-score). The I-score is a composite index of impulsivity [42], [43] and is calculated by subtracting the standard score of the mean latency to first response from the standard score of the total number of errors committed (Zerror-Zlatency). The E-score is calculated by summing the standard score of the mean latency to first response with the standard score of the total number of errors committed (1-(Zerror+Zlatency)).

#### Stop signal task

The stop signal task provides a measure of motor impulsivity. It requires subjects to make quick responses to visually presented go signals and to inhibit their response when a visual stop signal is suddenly presented. The current test is adapted from an earlier version of Fillmore and colleagues [44] and has been shown to be sensitive to stimulating as well as sedating drugs [3]. The go signals are four 1.5 cm letters (ABCD) presented one at a time in the center of a computer screen. Subjects are required to respond to each letter as quickly as possible by pressing one of two response buttons. One button is pressed to indicate that “A” or “C” appeared and the other to indicate “B” or “D”. Letters are displayed for 500 milliseconds and the computer screen is blank for 1.5 seconds before the next letter is displayed. This provides a period of 2 seconds in which the subject can respond to a letter. A single test consists of 176 trials in which each of the 4 stimuli is presented equally often. A stop signal occurs in 48 trials during the test. The stop signal consists of a visual cue, i.e. “*”, that appears in one of the four corners of the screen. Subjects are required to withhold their response in case a stop signal is presented. Stop signals are presented 12 times at each of the four delays after the onset of a letter: 50, 150, 250 and 350 milliseconds. Trials always begin with a 500 millisecond preparation interval in which a fixation cross appears at the center of the screen. Dependent variables are the proportion of commission errors on stop signal trials and the reaction times on go and stop signal trials (i.e. stop reaction time). Stop reaction time (SRT) to stop signal trials represents the estimated mean time required to inhibit a response.

The method for calculating stop reaction time was taken from the race model of inhibitory control [45]. This model proposes that the response to stop signal trials is defined by two parallel processes: execution of a motor action in response to a signal and inhibition of a motor action in response to a stop signal. Crucial to the outcome of the race is the speed of both processes. Response inhibition will fail if the time required to inhibit exceeds the time to complete a motor response at the time of the stop signal. The speed of the inhibition response cannot be observed directly but can be derived mathematically on the basis of three factors: stop-signal delay, reaction time distribution on go trials and the probability of successful response inhibitions in stop signal trials. First, reaction times to 128 go trials were rank ordered from shortest to longest. The finishing time of the inhibition response was then determined from the probability of successful response inhibition and the distribution of reaction times. If n-percent of the responses on stop-signal trials would be unsuccessfully inhibited (i.e. commission error), than the finishing time would be associated with the n-th percentile of the RT distribution. Stop reaction time was then determined by subtracting the appropriate stop-signal delay from reaction time at the n-th percentile of the RT distribution. The resulting values for each stop signal delay were then averaged to yield a single measure of stop reaction time for the test [3].

#### Cue-dependent reversal learning task

The cue-dependent reversal learning task is an adjusted form of the cue-dependent go-no-go task of Fillmore and Rush [46]. This test places emphasis on the anticipatory nature of inhibitory and activational mechanisms of control, which rapidly develop cue-dependence. It has also been shown to be highly sensitive to the effects of psychoactive drugs [47]. In this task, subjects are required to respond to target stimuli (Go) and to inhibit their response on non-target stimuli (No-Go). Stimuli are rectangles, appearing in the center of a computer screen, in a horizontal or vertical position. Targets are green and non-targets are blue rectangles. Cues provide preliminary information regarding the type of imperative target stimulus (i.e. Go or No-Go) that is likely to follow. The cues have a high probability of signaling the correct target. The ‘vertical rectangle’ precedes a Go-stimulus in 80% of the cases and a No-Go-stimulus in 20% of the cases. The ‘horizontal rectangle’ signals a No-Go in 80% of the cases and a Go-stimulus in 20% of the cases. This rule will be reversed a number of times throughout this task, depending on the performance of the subject. Subjects have to detect this rule-change and modify their response pattern. Subjects will be informed beforehand that the rule can change but not about the number of times this event will take place or when it is changed. This task measures the acquisition and discrimination-reversal learning of cue-target associations. More impulsive individuals have more problems inhibiting responses after a reversal of the rule than less impulsive individuals [47]. Dependent variables are number of correct responses and correct inhibitions.

**Table 1:** Mean (SE) values of summated scales on the POMS questionnaire, followed by a summary of main effects and interactions following 2 major GLM analyses.

GLM 1:											
	*Mean (± SE)*				*GLM (F; p)*						
Scale	Pla	Ketanserin	MDMA	Ketanserin x MDMA	Ketanserin		MDMA			Ketanserin x MDMA	
Anxiety	4.76 (.91)	4.82 (1.10)	8.06 (1.24)	7.06 (1.32)	–	–	10.97	.004		–	–
Depression	3.53 (1.76)	4.00 (2.03)	2.12 (.86)	6.71 (2.56)	5.46	.033	–	–		5.23	.036
Anger	2.47 (1.13)	3.88 (1.47)	2.71 (.59)	4.12 (1.25)	–	–	–	–		–	–
Vigor	10.47 (1.04)	8.35 (.99)	16.06 (1.50)	10.29 (1.39)	19.47	.000	14.12	.002		–	–
Fatigue	5.12 (1.45)	8.35 (1.45)	3.18 (1.00)	7.65 (1.49)	16.03	.001	–	–		–	–
Confusion	5.06 (.45)	6.53 (.98)	5.94 (.89)	8.23 (1.04)	30.83	.000	11.98	.003		–	–
Friendliness	15.71 (1.01)	15.71 (1.46)	21.65 (1.38)	16.94 (1.74)	6.71	.020	7.69	.014		5.41	.033
Elation	8.88 (.68)	8.41 (.86)	14.23 (1.33)	9.76 (1.17)	10.73	.005	21.48	.000		5.80	.028
Arousal	5.06 (2.67)	−1.71 (2.15)	15.00 (2.88)	1.47 (2.93)	42.09	.000	10.14	.006		–	–
Positive Mood	5.35 (1.85)	4.41 (2.16)	12.12 (1.93)	3.06 (2.96)	20.36	.000	–	–		11.35	.004
**GLM 2:**											
	***Mean (± SE)***				***GLM (F; p)***						
**Scale**	**Pla**	**Pindolol**	**MDMA**	**Pindolol x MDMA**	**Pindolol**		**MDMA**			**Pindolol x MDMA**	
Anxiety	4.76 (.91)	5.12 (.99)	8.06 (1.24)	8.35 (1.26)	–	–	12.28		.003	–	–
Depression	3.53 (1.76)	4.29 (2.10)	2.12 (.86)	5.06 (2.18)	–	–	–		–	–	–
Anger	2.47 (1.13)	3.94 (1.56)	2.71 (.59)	3.71 (1.45)	–	–	–		–	–	–
Vigor	10.47 (1.04)	9.94 (1.41)	16.06 (1.50)	15.29 (1.35)	–	–	13.93		.002	–	–
Fatigue	5.12 (1.45)	5.94 (1.22)	3.18 (1.00)	1.94 (.68)	–	–	10.59		.005	–	–
Confusion	5.06 (.45)	5.88 (.92)	5.94 (.89)	7.53 (.97)	7.00	.018	–		–	–	–
Friendliness	15.71 (1.01)	15.94 (1.60)	21.65 (1.38)	20.47 (1.28)	–	–	19.44		.000	–	–
Elation	8.88 (.68)	8.88 (1.00)	14.23 (1.33)	13.12 (1.10)	–	–	22.36		.000	–	–
Arousal	5.06 (2.67)	3.23 (2.75)	15.00 (2.88)	14.18 (2.20)	–	–	15.80		.001	–	–
Positive Mood	5.35 (1.85)	4.59 (2.29)	12.12 (1.93)	8.06 (2.74)	4.86	.042	18.25		.001	–	–

Significance (p<.05) and non-significance (−) is shown.

**Figure 2:**
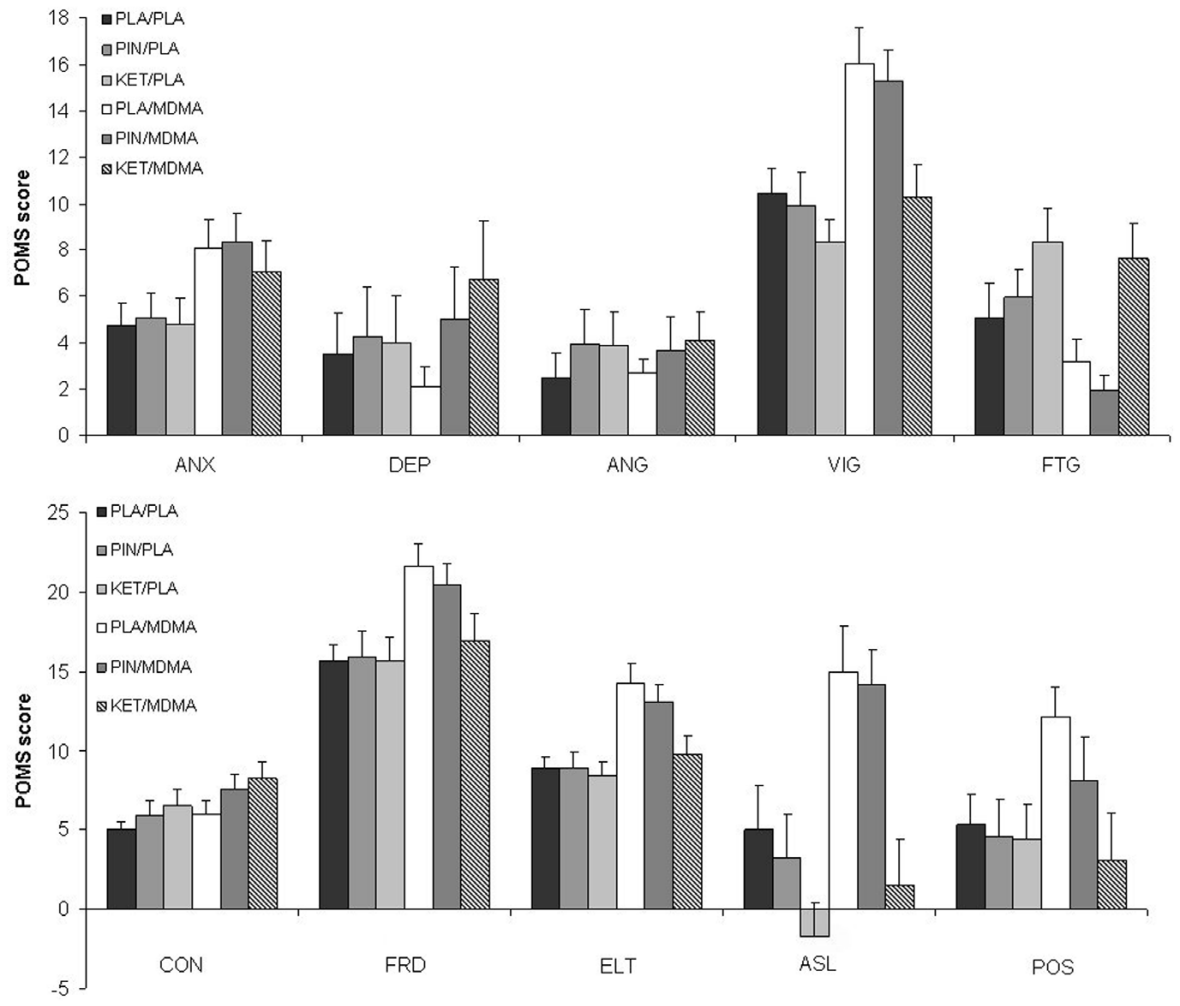
Mean (SE) ratings on the Profile of Mood States (POMS) questionnaire in every treatment condition (n = 17). ANX  =  anxiety, DEP  =  depression, ANG  =  anger, VIG  =  vigor, FTG  =  fatigue, CON  =  confusion, FRD  =  friendliness, ELT  =  elation, ASL  =  arousal, POS  =  positive mood.

### Blood Samples

Blood samples were collected before the start of the tasks at 1.5 hrs post T2. Blood samples were centrifuged immediately and the serum was subsequently frozen at −20°C until analyses for pharmacokinetic assessments. MDMA, pindolol and ketanserin concentrations were determined using solid phase extraction and gas chromatography with mass spectrometric detection.

### Statistics

The hypothesis that pretreatment with ketanserin or pindolol would interact with MDMA induced impulsivity and mood was tested in 2 separate General Linear Model (GLM) analyses. Impulsivity and mood effects of MDMA, Ketanserin and MDMA × Ketanserin were analyzed by means of a GLM repeated measures ANOVA with MDMA (2 levels, i.e. 75 mg MDMA and placebo) and Ketanserin (50 mg ketanserin and 50 mg ketanserin +75 mg MDMA) as the main factors. Impulsivity and mood effects of MDMA, Pindolol and MDMA × Pindolol were analyzed by means of a GLM repeated measures ANOVA with MDMA (2 levels, i.e. 75 mg MDMA and placebo) and Pindolol (20 mg pindolol and 20 mg pindolol +75 mg MDMA) as the main factors. In case of significant main effects, separate drug-placebo contrasts were conducted. The alpha criterion significance level was set at p = 0.05. All statistical tests were conducted with SPSS version 15.0.

**Table 2:** Mean (SE) scores and Summary of main effects and interactions following 2 major GLM analyses for all dependent variables in the matching familiar figures task (MFF20), the stop signal task (SST) and the cue-dependent reversal learning task.

GLM 1:	*Mean (± SE)*				*GLM (F; p)*									
Test	Pla	Ketanserin	MDMA	Ketanserin × MDMA	Ketanserin			MDMA				Ketanserin × MDMA		
*MFF20*														
Impulsivity (I)	0.02 (.93)	−.19 (.79)	.12 (1.04)	.13 (1.03)	–		–	–			–	–		–
Efficiency (E)	.02 (1.25)	−.19 (1.78)	.12 (1.41)	.13 (1.51)	–		–	–			–	–		–
Reaction time (s)	6.16 (1.27)	5.96 (1.22)	7.03 (1.49)	12.08 (3.55)	37.86		.000	62.37			.000	46.71		.000
*Stop signal Task (*n = 14*)*														
Stop Reaction Time (SRT)	255.07 (11.14)	289.64 (12.69)	277.86 (11.55)	309.71 (19.18)	10.34		.007	–			–	–		–
# Misses	10.57 (2.27)	11.93 (2.41)	12.36 (2.54)	11.93 (2.42)	–		–	–			–	–		–
# False Alarms	.79 (.64)	1.07 (.92)	.43 (.29)	3.86 (1.73)	–		–	–			–	–		–
Go-RT (ms)	600.97 (41.88)	628.94 (43.81)	611.41 (29.75)	652.06 (34.00)	4.80		.047	–			–	–		–
*Cue-dependent reversal learning task*														
# Correct	134.35 (.41)	134.76 (.14)	134.24 (.59)	132.88 (1.75)	–		–	–			–	–		–
# Correct inhibitions	133.41 (.34)	132.59 (.55)	133.24 (.70)	132.53 (.59)	7.55		.014	–			–	–		–
**GLM 2:**	***Mean (± SE)***				***GLM (F; p)***									
**Test**	**Pla**	**Pindolol**	**MDMA**	**Pindolol × MDMA**	**Pindolol**				**MDMA**				**Pindolol × MDMA**	
*MFF20*														
Impulsivity (I)	0.02 (.93)	−.12 (1.63)	.12 (1.04)	.03 (1.14)	–	–			–	–			–	–
Efficiency (E)	.02 (1.25)	−.12 (2.00)	.12 (1.41)	.03 (1.48)	–	–			–	–			–	–
Reaction time (s)	6.16 (1.27)	6.51 (1.08)	7.03 (1.49)	7.02 (1.40)	–	–			8.46	.010			–	–
*Stop signal Task (*n = 14*)*														
Stop Reaction Time (SRT)	255.07 (11.14)	253.71 (10.10)	277.86 (11.55)	274.57 (14.94)	–	–			9.56	.009			–	–
# Misses	10.57 (2.27)	11.14 (2.37)	12.36 (2.54)	11.79 (2.9)	–	–			–	–			–	–
# False Alarms	.79 (.64)	1.36 (1.13)	.43 (.29)	.57 (.50)	–	–			–	–			–	–
Go-RT (ms)	600.97 (41.88)	596.54 (31.24)	611.41 (29.75)	605.56 (36.13)	–	–			–	–			–	–
*Cue-dependent reversal learning task*														
# Correct	134.35 (.41)	133.41 (.82)	134.24 (.59)	134.82 (.095)	–	–			–	–			–	–
# Correct inhibitions	133.41 (.34)	132.11 (.46)	133.24 (.70)	133.71 (.47)	–	–			–	–			11.59	.004

Significance (p<.05) and non-significance (−) of main effects is shown.

## Results

Seventeen complete data sets entered statistical analysis, except for the Stop signal task (N = 14), where 3 subjects were excluded due to technical or performance failures during test administration.

### Subjective Measures

#### POMS

A summary of GLM statistics for all POMS scales is given in Table 1. Mean (SE) subjective ratings on the POMS scales in every treatment condition are shown in Figure 2.

MDMA significantly increased feelings of anxiety, confusion, vigor, friendliness, elation, positive mood and arousal. The effect of MDMA on positive mood was highly significant (p<.001) in the GLM model including pindolol, and almost reached significance (p = 0.057) in the GLM model including ketanserin. In addition, MDMA also significantly reduced feelings of fatigue. Pindolol significantly increased feelings of confusion and decreased positive mood. There was no significant interaction between pindolol and MDMA. Ketanserin affected almost all subscales of the POMS. It significantly increased feelings of depression, fatigue and confusion and decreased feelings of vigor, friendliness, elation, arousal and positive mood. The interaction between MDMA and ketanserin reached significance on 4 POMS subscales: depression, friendliness, elation and positive mood. After the combination of MDMA and ketanserin, ratings of friendliness, elation and positive mood returned to placebo levels, whereas feelings of depression increased.

### Impulsivity Tasks

The mean (SE) performance on impulsivity tasks for every treatment condition and a summary of GLM statistics for all impulsivity parameters is given in Table 2.

MDMA significantly increased SRT in the stop signal task and RT in the MFF20 but generally failed to affect performance on any of the other impulsivity parameters. Pindolol did not affect any impulsivity parameters. Ketanserin increased SRT and RT in the stop signal task and decreased the number of inhibitions in the cue-dependent reversal learning task. There were no significant interactions between MDMA and ketanserin. There were also no interactions between MDMA and pindolol other than an increase in correct inhibitions in the cue-dependent reversal learning task.

### Blood Samples

Mean (SD) MDMA concentration in serum at 1.5 hours after MDMA administration was 157 (48) ng/mL. When combined with ketanserin or pindolol, mean (SD) serum concentration was 164 (62) and 156 (56) ng/mL respectively. Mean (SD) serum concentrations of ketanserin and pindolol were 86 (42) and 133 (80) ng/mL respectively. In the condition where ketanserin or pindolol was combined with MDMA, the mean (SD) serum concentrations of both drugs were 104 (41) and 130 (53) ng/mL respectively.

## Discussion

The aim of the current study was to investigate the role of 5-HT_1_ and 5-HT_2_ receptors in MDMA induced changes in mood and impulsive behavior. Single doses of MDMA significantly increased positive as well as negative moods as rated with the POMS questionnaire. MDMA raised feelings of vigour, friendliness, elation and arousal, while also making subjects feel more anxious and confused. These findings are in line with previous studies that also reported a marked effect, both positive and negative, of MDMA administration on mood ratings [1], [2], [12], [19], [26], [48], [49]. Single doses of MDMA increased SRT in a stop signal task and reaction time in the MFF20 indicating a slowing of inhibitory and reflective responses during these tasks. Other measures of impulsivity did not show any effects of MDMA. In general however, acute effects of MDMA on mood and impulsivity were sufficiently present to assess the contributing roles of 5-HT_1_ and 5-HT_2_ receptors during pretreatments with ketanserin and pindolol.

Pretreatment with ketanserin significantly interacted with MDMA on the subscales representing positive moods (friendliness, elation and positive mood). Blockade of 5-HT_2_ receptors with ketanserin basically prevented MDMA to affect positive moods at all. POMS ratings of positive mood during the combination of ketanserin and MDMA were similar to the ratings during placebo. Ketanserin alone also significantly decreased positive mood rating. The magnitude of these effects was very small relative to the increase in positive moods produced by MDMA. Consequently, combined effects of ketanserin and MDMA cannot be explained as a summation of drug effects produced by MDMA and ketanserin separately, but truly indicates a drug interaction indicating that blockade of 5-HT_2_ receptors also blocks MDMA effects on positive moods. Pretreatment with ketanserin however did not reverse MDMA induced anxiety and the combination increased ratings of depression. This finding strongly indicates that the 5-HT_2_ receptor is only involved in mediating positive moods during intoxication and is not involved in some of the negative moods produced by MDMA.

Pretreatment with pindolol did not interact with the effects of MDMA on mood. When given alone, pindolol produced small but significant increments in feelings of confusion and small decrements in positive mood. The lack of interaction between MDMA and pindolol illustrated that the 5-HT_1_ receptor does not play a role in mediating MDMA induced mood states. The finding is in line with a previous mechanistic study [50] showing that pretreatment with pindolol does not affect MDMA induced moods. Alternatively, one could also argue that pindolol blocks only 40% of 5HT_1A_ receptors [34] and that this may not suffice to measurably attenuate any 5HT_1A_ mediated MDMA effects. We cannot exclude this possibility, but unfortunately, alternative 5HT_1A_ ligands that fully block 5HT_1A_ receptors are presently not available.

None of the pretreatments interacted with the effects of MDMA on measures of impulsivity. It should be noted, however, that the effects of MDMA were limited to an increment of SRT in the stop signal task and reaction time in the MFF20 and did not affect other measures of impulsivity. Previous studies have also shown a mix of either positive or neutral effects of single doses of MDMA on impulsivity [1], [2], [16], [18], [19]. Possibly, the lack of MDMA effects on most measures of impulsivity may be related to the fact that these measures represent different psychological and neuropharmacological constructs of impulse control [51]. Impulsivity is not a unitary, one-dimensional construct but can encompass different types of impulsivity [52], [53], [54]. Two types of impulsivity that can be distinguished are cognitive impulsivity and motor impulsivity. Cognitive impulsivity, as measured by the MFF20, is believed to reflect complex processes involved in the control of several cognitive, behavioral and effective processes. Motor impulsivity or response inhibition as measured by the stop signal task and the cue dependent reversal learning task, on the other hand, is believed to relate to the executive control of motor processes only [55]. Thus, it is possible that MDMA affects only a subset of processes related to motor impulsivity and cognitive impulsivity but leaves other subsets unaffected [3]. Alternatively, the relationship between MDMA and impulsivity may also be marginal and not a key feature during MDMA intoxication.

In conclusion, results from the current study show that administration of MDMA has both positive and negative influences on mood states. Furthermore, pretreatment with a 5-HT_2_ receptor antagonist affects MDMA-mediated responses on a number of positive subscales of the POMS, suggesting that the 5-HT_2_ receptor might be involved in mediating positive mood states. On the contrary, treatment with a partial 5-HT_1_ receptor antagonist did not interfere with MDMA effects on mood. Blockade of 5-HT_1_ and 5-HT_2_ receptors did not interact with the effects of MDMA on measures of impulse control.
